# Glioma SOX2 expression decreased after adjuvant therapy

**DOI:** 10.1186/s12885-019-6292-y

**Published:** 2019-11-12

**Authors:** Wei Yu, Xiaoqiu Ren, Chunxiu Hu, Yinuo Tan, Yongjie Shui, Zexin Chen, Lili Zhang, Jiaping Peng, Qichun Wei

**Affiliations:** 1 Department of Radiation Oncology, the Second Affiliated Hospital, Zhejiang University School of Medicine, Jiefang Road 88, Hangzhou, 310009 People’s Republic of China; 2 Cancer Institute (Ministry of Education Key Laboratory of Cancer Prevention and Intervention), Zhejiang University Cancer Institute, Hangzhou, 310009 People’s Republic of China; 3 Department of Radiation Oncology, Zhejiang Quhua Hospital, Quzhou, 324000 People’s Republic of China; 4 Department of Medical Oncology, the Second Affiliated Hospital, Zhejiang University School of Medicine, Hangzhou, 310009 People’s Republic of China; 5 Center of Clinical Epidemiology and Biostatistics for statistical analysis, the Second Affiliated Hospital, Zhejiang University School of Medicine, Hangzhou, 310009 People’s Republic of China

**Keywords:** Glioma, Recurrence, Prognosis, SOX2

## Abstract

**Background:**

SOX2 is regarded as an important marker in stem cell. The change of SOX2 expression after adjuvant therapy in high grade glioma (HGG) remains unknown so far. Few patients with recurrent glioma have opportunity to undergo operation once again, so the recurrent glioma samples are scarce. This study tries to analyze SOX2 expression in paired primary and recurrent HGG, aims to better understand the transformation law of SOX2 after adjuvant therapy in HGG.

**Methods:**

Twenty-four recurrent HGG patients who undergone a second resection were included. 16 patients received adjuvant therapy, the remaining 8 patients didn’t receive any adjuvant therapy at all. The protein expression of SOX2 in paired primary and recurrent HGG was tested by immunohistochemistry. The statistical analysis was conducted by IBM SPSS Statistics 19.0.

**Results:**

In primary HGG, SOX2 expression of 3 + , 2 + , 1+ and 0+ were seen in 20 (83.3%), 1 (4.2%), 1 (4.2%) and 2 cases (8.3%), respectively. The expression of SOX2 was decreased in recurrent HGG compared to the paired primary sample (*p* = 0.001). The decrease of SOX2 was often seen in patients received chemotherapy, radiotherapy or both (*p* = 0.003). Patients with SOX2 high expression in primary glioma had a longer median PFS than those with SOX2 low expression with marginal statistic significance (12.7 vs. 5.4 months, *p* = 0.083). For cases with SOX2 high expression in the primary glioma, those had SOX2 low expression after recurrence seemed to have worse prognosis as compared to patients with stable SOX2 high expression (PFS: 10.4 vs. 14.9 months, *p* = 0.036; OS: 27.0 vs 49.5 months, *p* = 0.005).

**Conclusions:**

This is the first study comparing the protein expression of SOX2 in recurrent HGG and its paired primary tumor. SOX2 high expression is common in brain HGG, a tendency of decreased SOX2 expression in recurrent gliomas was evidenced. Lower SOX2 expression was seen in those patients who received adjuvant chemotherapy and/or radiotherapy. Patients with low SOX2 expression in primary HGG usually have poorer prognosis, those with SOX2 expression decreased in recurrent HGG had worse outcome.

## Background

Glioma is the most common primary intracranial tumor, and it’s usually subdivided into WHO I to IV grades [1]. Grade I-II gliomas are considered as low grade gliomas (LGGs). The prognosis of LGGs is relatively better with a 5 to 10 years’ median overall survival (OS) [2–5]. However, 50–75% patients with LGGs would die of disease progression or deteriorate into higher grade gliomas [4]. Grade III-IV are called high grade gliomas (HGGs). HGGs are characterized by high recurrence rate and dismal survival. WHO IV grade glioma, named glioblastoma multiforme (GBM), occupy the highest proportion in glioma, with the highest malignant degree and poorest prognosis [1, 6]. Currently, standard care for newly diagnosed glioblastoma is maximum safe resection of the malignancy followed by radiotherapy (RT) and temozolomide (TMZ) chemotherapy. Grave median survival of GBM was reported as 14.6 months despite state-of-the-art treatment [7]. Almost all glioblastoma recurred inevitably.

SOX2 (SRY-related HMG-box 2) is a key member of transcription factor SOX family. It is mainly expressed in embryonic and adult stem cells, including neural progenitor cells, etc. It can also be expressed in tumor stem cells, such as glioma stem cells. SOX2 can stimulate somatic cell transform into pluripotent stem cell combined with other stem cell markers, such as NANOG and OCT, and also maintain the characteristics of cancer stem cells. It is a glioma stem cell marker [8]. The expression of SOX2 is associated with tumor formation, chemotherapeutic resistance and the tumor stem cell like phenotype [9, 10]. Schmitz and other studies have found that SOX2 was overexpressed in malignant gliomas and its expression in normal brain tissue was rare [11]. Gangemi et al. reported that silencing the SOX2 gene in tumor initiating cells of glioma can prevent cell proliferation, resulting in the loss of tumorigenicity in immunodeficient mice [12]. Ikushima et al. showed that inhibition of TGF-beta signaling can significantly reduce the tumorigenesis of glioma initiating cells by promoting differentiation, and this effect attenuated in SOX2 transduced tumor initiating cells [13]. Therefore, SOX2 gene is the key gene for the maintenance of the characteristics of glioma stem cells.

Few patients have opportunity to undergo operation once again. The data used to compare the primary glioma and its paired recurrent tumor is scarce. SOX2 expression of recurrent gliomas remains unknown so far. In this study, we collected 24 cases with paired sample of primary HGGs and corresponding recurrent tumor after a second resection. We attempt to analyze the expression of SOX2 protein of primary HGG and its paired recurrent tumor in order to better understand the biological behaviour of HGG and the transformation law of SOX2 in primary and recurrent HGG, and eventually enrich our kownledge of HGG.

## Methods

### Patients

This study was consented by the Institutional Review Board of the Second Affiliated Hospital, Zhejiang University School of Medicine (SAHZU). Informed consent was obtained from all the patients before surgery regarding the data and samples to be used for research. Patients had undergone first operation for HGG at SAHZU from Jan. 2008 to Dec. 2014 were retrospectively analyzed, 24 recurrent patients with second surgery were included. Histopathology diagnosis were achieved after surgery and diagnosed by neuropathologists. The medical records were retrospectively reviewed.

### Immunohistochemistry

Formalin-Fixed and Parrffin-Embedded (FFPE) blocks from the pathology department of SAHZU were cut into serial 4 um slices with a microtome. The paraffin sections were incubated in 60 °C in incubator overnight.. Then the sections were deparaffinized in xylene and rehydrated through graded alcohols (100, 95, 75%). The sections were put in boiled antigen retrieval solution (EDTA, PH = 9) in electric cooker for 10 min and heat preserved for 10 min. Then the sections were incubated with 100ul SOX2 primary antibody (ZSGB-BIO, China) at room temperature for 2.5 h. After removing the primary antibody, the sections were incubated with 100ul ready-to-use secondary antibody (Polink-1 HRP staining system (ZSGB-BIO)) at room temperature for 2.5 h. After removing the secondary antibody, the sections were incubated with chromagen 3, 3′-diaminobenzidine (DAB) at room temperature for 1.5 min. The sections were counterstained with hematoxylin for 3 min and dehydration through graded alcohols (75, 95, 100%). Then the slides were covered slips with mounting medium.

### Scoring system

Immunohistochemical staining evaluation of SOX2 protein was based on the range of staining. The proportion of immunopositive cells among the total number of tumor cells was subdivided into 4 categories, as follows: 0: < 10%; 1: 10–50%; 2: 50–90%; 3: > 90% [14]. Two pathologists read the pathological sections and were blinded to the clinical information and histological grades.

### Statistical analysis

Chi-square test and Fisher’s exact test were used among subgroups to compare enumeration data such as sex, WHO grade, tumor location, adjuvant therapy, residue, and type of recurrence. *P* < 0.05 was considered significant. For the ranked data of paired primary and recurrent samples, Wilcoxon rank sum test was used to analyze the variation trend of SOX2 expression. Correlation between SOX2 expression and WHO classification were tested by Spearman rank correlation test. Overall survival (OS) is defined as the time from the first operation of glioma to the death of the patient. Progression-free survival (PFS) is defined as the time from the first operation of glioma to the progression of the tumor. Kaplan Meier method and Cox proportion hazard regression model were used to estimate PFS and OS for univariate and multivariate analysis. *P* < 0.05 was statistically significant. IBM SPSS Statistics 19.0 was used for statistical analyses.

## Results

### Patient clinical characteristics

24 HGG patients with pathologically confirmed and complete data who received second operation when recurred after first operation were included. There were 16 males and 8 females. The average age at first operation was 45.4 years (range: 12–60 years old). Seventeen cases had gliomas in frontal lobe, 5 in temporal lobe, 1 in parietal lobe and 1 in insula lobe. At the first operation, 14 patients underwent subtotal resection and the other 10 cases total gross resection. After the first operation, 12 cases were diagnosed as grade III gliomas, 12 cases were glioblastomas. 22 cases were IDH1 wild type, 2 cases were IDH1 mutant type which were both SOX2 high expression, their PFS and OS were both longer than the medium level (PFS: 20 & 13.5 months; OS: 49.5 & 57.0 months). Twelve patients received radiotherapy and chemotherapy after the first operation, 3 patients only received radiotherapy, 1 patient only received chemotherapy, and the rest 8 patients had not adjuvant therapy at all.

At the second operation for recurrent tumors, 18 patients underwent subtotal recection and 6 cases total gross recection. In recurrent gliomas, 6 cases were grade III gliomas, 18 cases were glioblastomas. Six cases deteriorated from grade III gliomas to glioblastomas. After the operations for recurrent tumors, 4 cases received both radiotherapy and chemotherapy, 1 patient only received radiotherapy, 7 patients chemotherapy alone, and the rest 12 cases had no more adjuvant therapy.

The deadline for follow-up was August 18, 2017. The overall median PFS was 12.7 months (95% CI: 10.0–15.4 months): 14.9 months (95% CI: 9.0–20.8 months) for the primary grade III gliomas and 10.3 months (95% CI: 7.5–13.0 months) for the primary glioblastomas. At the end of the follow-up visit, 4 patients were alive. The median OS was 30 months (95% CI: 22.2–37.8): 31.0 months (95% CI: 26.6–35.4) for the primary grade III gliomas and 24.4 months (95% CI: 22.2–37.8 months) for the primary glioblastomas. The median OS after second operation was 18.1 months (95% CI: 13.2–23.0).

### SOX2 expression in primary glioma

**Fig. 1:**
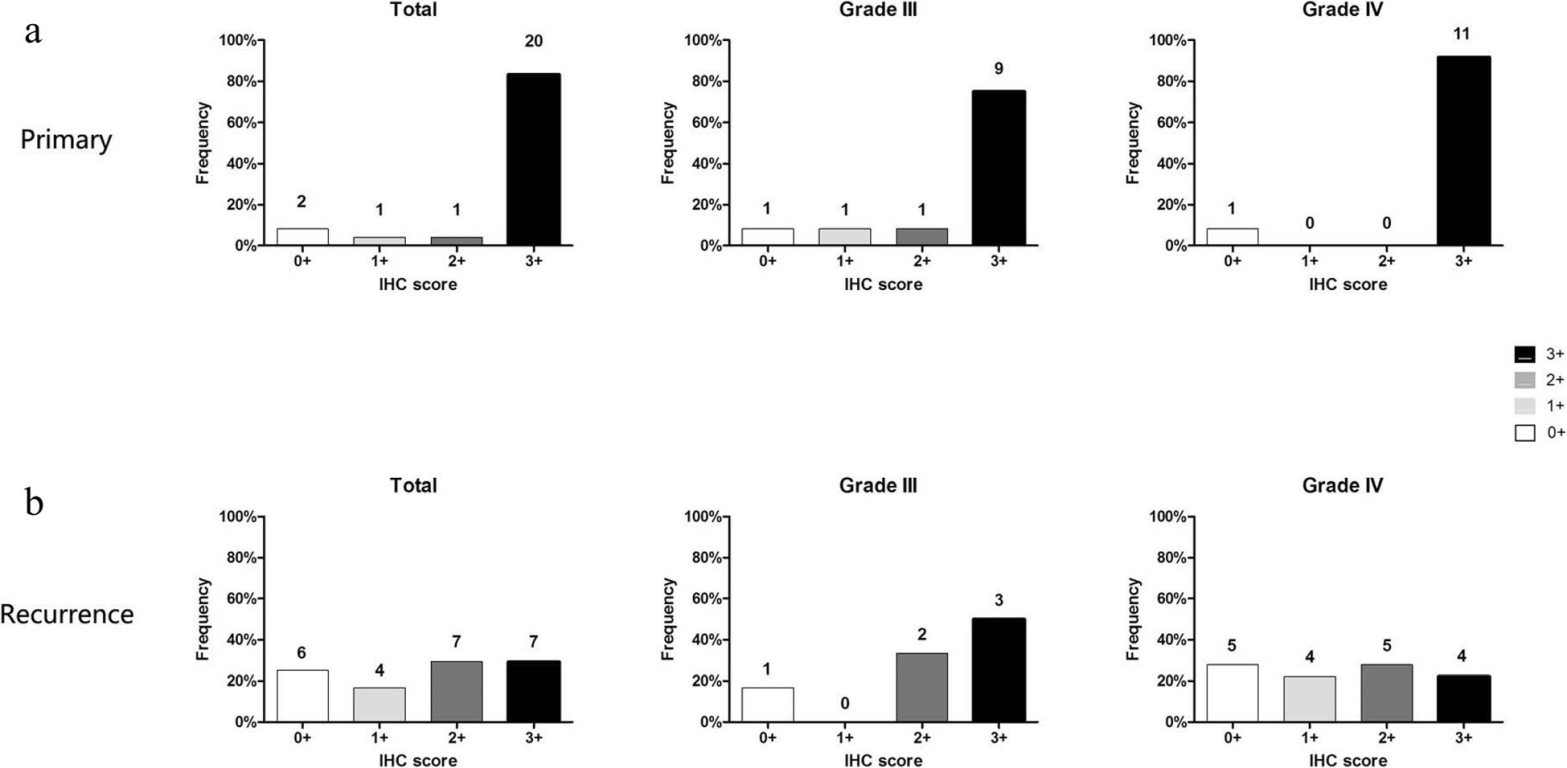
Expression of SOX2 in primary and recurrent HGGs. **a** Expression of SOX2 in primary glioma in the cohorts of totality, III and IV glioma. **b** Expression of SOX2 in recurrent glioma in the cohorts of totality, III and IV glioma

In primary HGGs, SOX2 expression of 3 + , 2 + , 1+ and 0+ were seen in 20 (83.3%), 1 (4.2%), 1 (4.2%) and 2 cases (8.3%), respectively. The corresponding data for grade III glioma were 9 (75.0%), 1 (8.3%), 1 (8.3%) and 1 cases (8.3%), and for glioblastoma were 11 (91.7%), 0 (0.0%), 0 (0.0%) and 1 cases (8.3%) (Fig. 1).

### Decreased SOX2 protein expression in recurrent gliomas compared with their paired primary tumors

The overall expression of SOX2 decreased (*p* = 0.001). In recurrent HGGs, SOX2 expression of 3 + , 2 + , 1+ and 0+ were seen in 7 (29.2%), 7 (29.2%), 4 (16.7%) and 6 cases (25.0%), respectively. The corresponding data for grade III gliomas were 3 (50.0%), 2 (33.3%), 0 (0.0%) and 1 cases (16.7%), and for glioblastomas were 4 (22.2%), 5 (27.8%), 4 (22.2%) and 5 cases (27.8%) (Fig. 1).

**Fig. 2:**
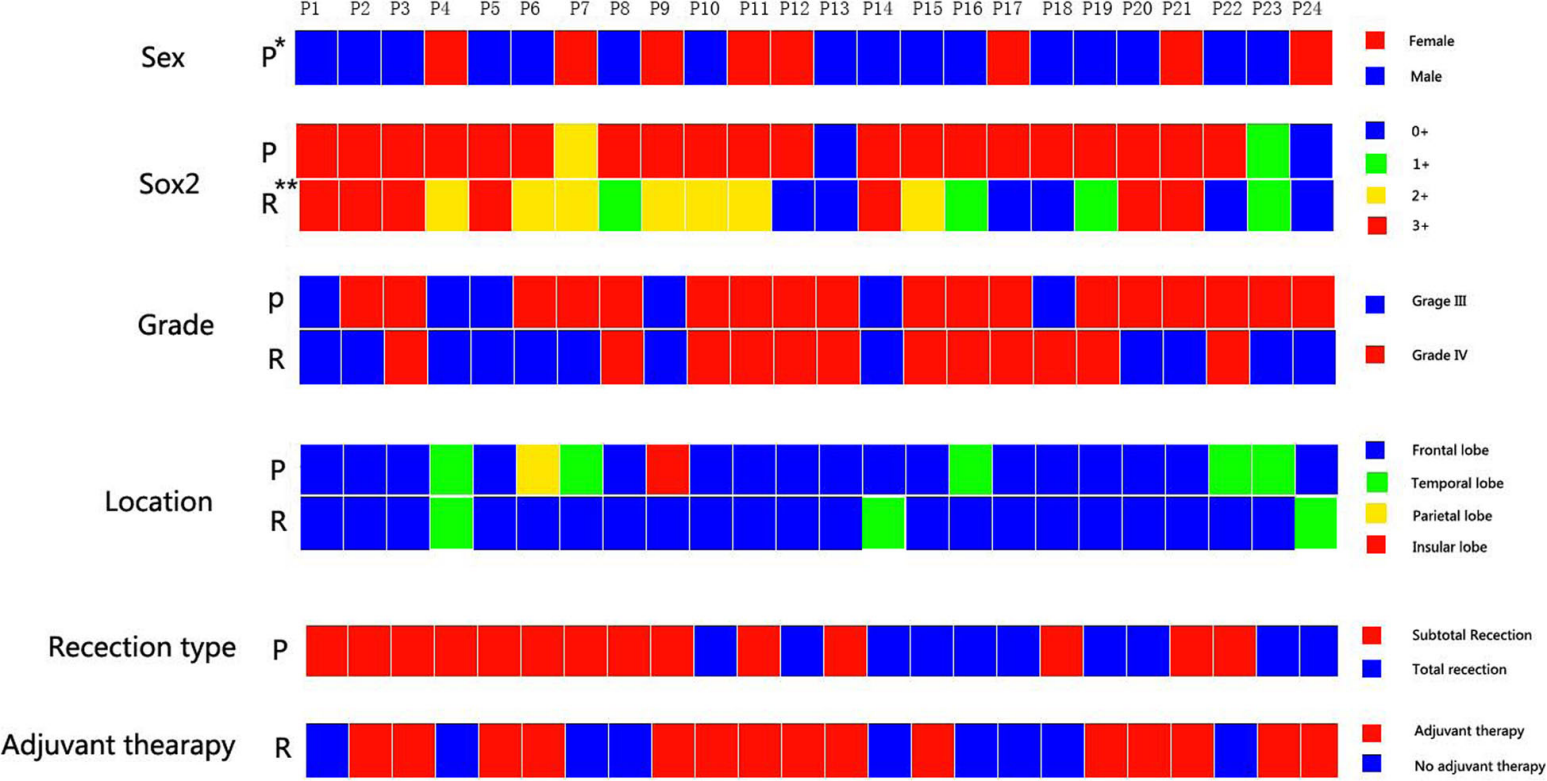
Change in SOX expression from primary to recurrent glioma with different layer of information including grade, treatment, sex, tumor location and resection type. * P=Primary; ** R = recurrence

**Fig. 3:**
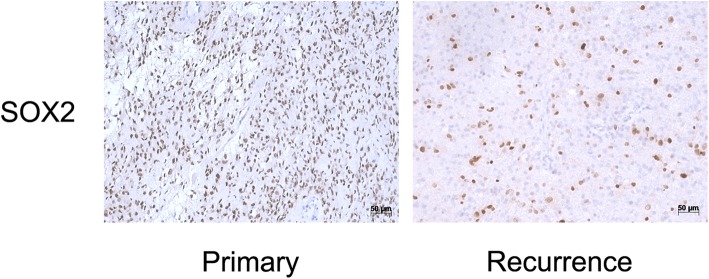
Expression of SOX2 in a representative patient with primary and paired recurrent gliomas. The patient was diagnosed as glioblastoma with SOX2 3+ after first operation in 2013, the histological diagnosis was stilll glioblastoma when recurrence in 2014, but the expression of SOX2 was negative (0+)

Compared with primary high grade tumors, SOX2 expression increased in their paired recurrent gliomas in 0 cases (0.0%), while 13 cases (54.2%) decreased, 11 cases (45.8%) did not change. In patients with grade III in primary gliomas and developed into glioblastoma in recurrent gliomas, 1 cases (1/6, 16.7%) with SOX2 expression decreased and 5 cases (5/6, 83.3%) were stable. In patients with grade III in primary and paired recurrent gliomas, 2 cases with SOX2 expression decreased (2/5, 40.0%) with SOX2 expression decreased, 3 cases (3/5, 60.0%) were stable. In patients with glioblastoma in primary and paired recurrent gliomas, 10 cases (10/11, 90.9%) with SOX2 expression decreased, 1 cases (1/11, 9.1%) were stable (Fig. 2). Figure 3 show the expression of SOX2 in primary and its paired recurrent glioma in a typical patient.

### Effect of postoperative adjuvant therapy on the expression of SOX2

Out of the 24 patients, 16 patients received adjuvant therapy including radiotherapy, chemotherapy and chemoradiotherapy after first surgery (adjuvant group), the other 8 patients received no adjuvant therapy at all (non-adjuvant group). Compared with primary gliomas, the overall expression of SOX2 decreased in adjuvant group (*p* = 0.003), but such tendency was largely not seen in non-adjuvant group (*p* = 0.317) (Table 1).

**Table 1:** Effect of postoperative adjuvant therapy on the expression of SOX2

Group(n)	Primary (%)					Recurrence (%)			
	0+	1+	2+	3+	0+	1+	2+	3+	P ^a^
No adjuvant therapy *n* = 8	1	0	1	6	1	1	2	4	0.317
Adjuvant therapy *n* = 15	1	1	0	14	5	3	5	3	0.003
Chemo-radiotherapy *n* = 12	1	1	0	10	5	2	3	2	
Radiotherapy *n* = 3	0	0	0	3	0	1	1	1	
Chemotherapy *n* = 1	0	0	0	1	0	0	1	0	

^a^Wilcoxon rank sum test was used to analyze the change of SOX2 expression between paired primary and recurrent samples

### SOX2 expression correlates with survival of Glioma

**Fig. 4:**
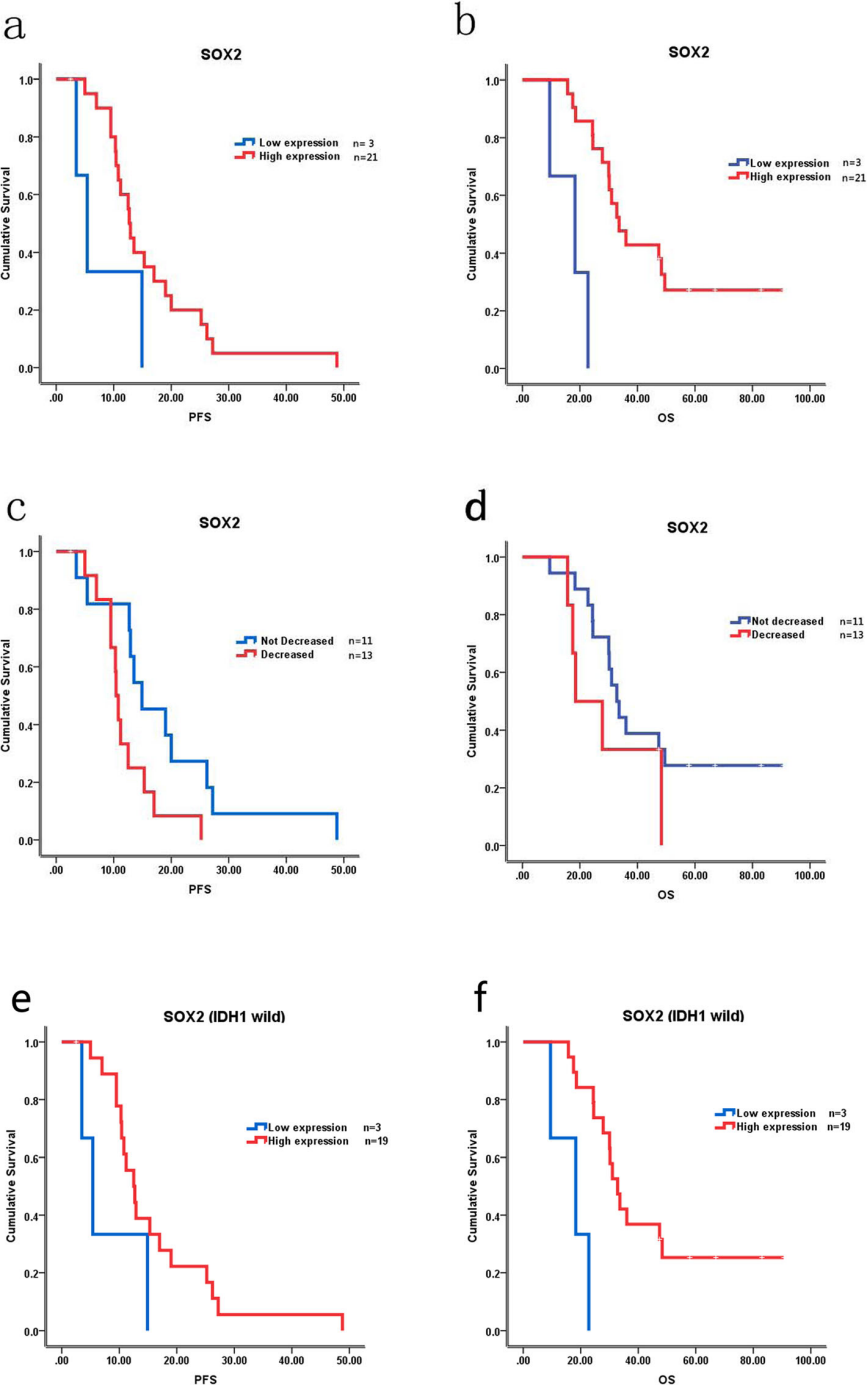
Correlation between the expression of SOX2 and prognosis. **a** Correlation between the expression of SOX2 and PFS. **b** Correlation between the expression of SOX2 and OS. **c** Correlation between the decreasing expression of SOX2 and PFS in patients with SOX2 high expression in primary HGG. **d** Correlation between the decreasing expression of SOX2 and OS in patients with SOX2 high expression in primary HGG. **e** Correlation between the expression of SOX2 and PFS in primary glioma with IDH1 wild type. **f** Correlation between the expression of SOX2 and OS in primary glioma with IDH1 wild type

Patients were grouped into SOX2 high expression group (2+ and 3+, *n* = 21) and SOX2 low expression group (0+ and 1+, *n* = 3) according to the expression of SOX2 in primary gliomas. The comparison of baseline clinical characteristics between those 2 groups was seen in Table 2. Univariate analysis showed that the median PFS was longer in SOX2 high expression group than in the SOX2 low expression group (12.7 vs. 5.4 months, *p* = 0.083), but *p* value had no significant difference (Fig. 4a). The median OS was also longer in SOX2 high expression group than in the SOX2 low expression group with significant difference 33.6 vs. 18.3 months, *p* < 0.001) (Fig. 4b). In patients with SOX2 high expression in primary glioma, 13 cases (54.2%) changed to SOX2 low expression after recurrence, the prognosis of these patients seemed worse than patients with stable SOX2 expression (PFS: 10.4 vs. 14.9 months, *p* = 0.036; OS: 18.5 vs 32.8 months, *p* = 0.249) (Fig. 4c, d). In patients with IDH1 wild type, the median PFS and OS was also longer in SOX2 high expression group than in the SOX2 low expression group (PFS: 12.5 vs 5.4, *p* = 0.098; OS: 32.8 vs 18.3, *p* = 0.001) (Fig. 4e, f). Multivariate analysis showed that SOX2 was an independent prognostic factor (Table 3).

**Table 2:** Comparison of baseline clinical characteristics between SOX2 low expression and high expression group in primary glioma

Characteristics	SOX2 high expression(*n* = 21)	SOX2 low expression(*n* = 3)	*p* value
Age (year)			
Mean (range)	45(22–60)	49(43–53)	0.373
Sex			
Male	14	2	
Female	7	1	1.000
WHO grade			
III	10	2	
IV	11	1	0.546
IDH1 status			
Wild type	19	3	
Mutated	2	0	1.000
Resection type			
Total gross recection	8	2	
Subtotal recection	13	1	0.55
Adjuvant therapy after first surgery			
Radiotherapy	3	0	
Chemotherapy	1	0	
Chemoradiotherapy	10	2	
No adjuvant therapy	7	1	0.266
Type of recurrence			
Local recurrence	19	1	
Distant recurrence	2	1	0.343
Median PFS (month)	12.7	5.4	0.083
Median OS (month)	33.6	18.3	< 0.001

**Table 3:** Results of multivariate analyses with the Cox proportional hazard models

Variable	Overall survival		Progression-free survival	
	HR(95% CI)	P	HR(95% CI)	P
Age	1.060	0.08	1.000	1.000
Sex	0.641	0.438	3.070	0.122
Adjuvant therapy after first surgery	1.121	0.852	1.459	0.563
Resection type	1.171	0.799	0.460	0.244
SOX2	0.215	0.076	0.160	0.045
WHO grade	1.722	0.427	11.606	0.001
Type of recurrence	2.301	0.377	1.166	0.866

**Fig. 5:**
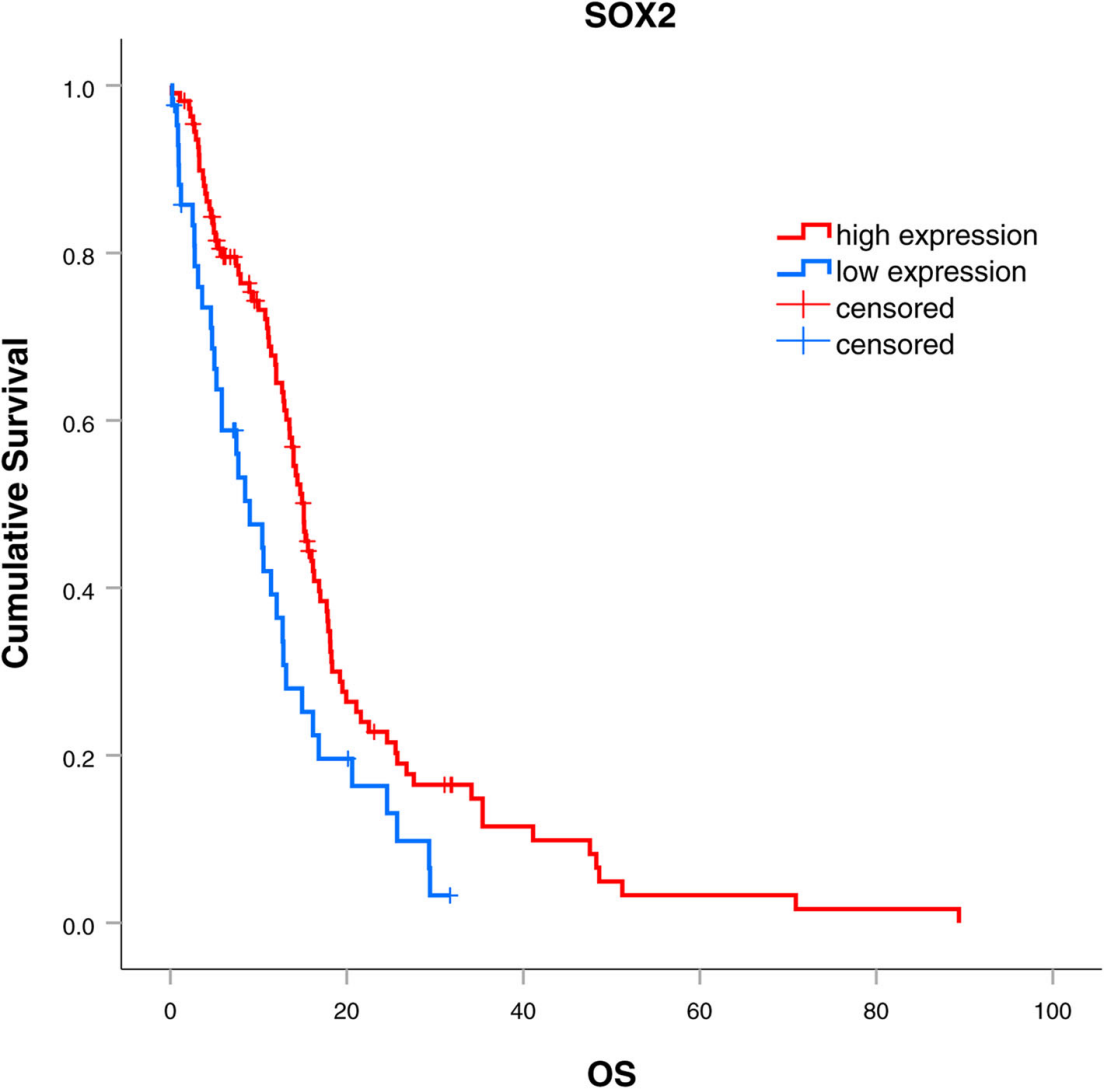
Correlation between the mRNA expression of SOX2 and OS in TCGA database

For the small sample size to analyze the prognostic value of SOX2, we further searched The Cancer Genome Atlas (TCGA) database and found SOX2 mRNA expression in 153 glioma cases (Additional file 1: Table S1). SOX2 low expression also predicted poor survival (Fig. 5).

## Discussion

This is the first study comparing the protein expression of SOX2 in recurrent HGG and its paired primary tumor. SOX2 high expression is common in brain gliomas, a tendency of decreased SOX2 expression in recurrent HGG was evidenced. Lower SOX2 expression was seen in those patients who received adjuvant chemotherapy and/or radiotherapy. Patients with low SOX2 expression in primary HGG usually have poorer prognosis, those with SOX2 expression decreased in recurrent glioma had worse outcome.

In our case series, about 83.3% of the primary HGG cases were SOX2 high expression. Elsir et al. [14] and Ballester et al. [15] reported a SOX2 high expression rate of 97.8 and 43.5% in primary HGGs. In the study by Guo et al. [16], western blot and RT-PCR were performed to evaluate the expression of SOX2, 95% of the gliomas expressed SOX2 at both the mRNA and protein levels. The results in our study are inconsistent with the previous reports.

The protein expression of SOX2 in recurent glioma has not been reported. For the first time, we presented that SOX2 expression decreased in recurrent glioma as compared to the corresponding primary glioma. It has been known that among proneural, mesenchymal and proliferative subtypes, the prognosis of the proneural subtype is better than the other two subtypes [17]. Verhaak et al. found that SOX2 expression was mainly in the proneural subtype and was rarely expressed in the mesenchymal and proliferative subtypes [18]. While the recurrent glioma tended to transform into the mesenchymal subtype [19, 20]. Wang et al. found that low miR-21/high SOX2 group tended to express in pre-neuronal and classical genotypes, while most of the high miR-21/low SOX2 group belongs to mesenchymal phenotype [21]. Therefore, decreased SOX2 expression in recurrent glioma might probably due to the tumor transformation from the proneural subtype into the mesenchymal subtype.

To study the influence of adjuvant therapy on the expression of SOX2, we further conducted subgroup analysis according to the adjuvant therapy patients received after first operation. A novel finding was that patients received adjuvant chemotherapy/radiotherapy usually had decreased SOX2 expression in the recurrent tumor. While those without adjuvant therapy had largely the same SOX2 expression in the recurrence as compared to the primary tumor.

This study indicated that low expression of SOX2 in primary HGG is a negative prognosticator although with marginal statistic significance. The prognostic value of SOX2 in HGG remained controversial so far. Sathyan et al. found that glioblastoma patients with low miR-21/high SOX2 expression had longer overall survival than those with high miR-21/low SOX2 (15.3 vs. 16.7 months, *p* = 0.0088), which was in accordance with our results. While Wang et al. reported that expression of SOX2 predicted poor prognosis [22]. It has been reported that expression of SOX2 related to promoter methylation [23–29]. This might allow the transposition of SOX2 protein testing to DNA methylation testing, which could be performed in cerebral spinal fluid, in less invasive mode, using cell free circulating DNA. Though testing SOX2 methlation status from cerebral spinal fluid, we can predicted the prognosis of the patients and choose the appropriate treatment strategy.

In the primary SOX2 high expression gliomas, 2 cases had IDH1 mutation, their PFS and OS were both longer than the medium level (PFS: 20 & 13.5 months; OS: 49.5 & 57.0 months). While the primary SOX2 low expression cases were IDH1 wild. It has been reported that IDH1 mutation predicted better prognosis in glioma [30, 31]. In these two cases, IDH1 mutation status and SOX2 expression were consistent to predict better prognosis. To elimilate the influence of IDH1 to the prognosis, we further analyzed the the correlation between SOX2 expression and prognosis in IDH1 wild type group. Finally, we found SOX2 low expression still had worse PFS and OS than SOX2 high expression group.

Twenty four HGG cases with both primary tumor samples and recurrent samples were analyzed in the present study. It is not a large patient series. However, few patients with recurrent glioma have opportunity to undergo operation once again, it is not easy to get the paired samples. Our study provides precious knowledge about SOX2 status in the recurrent gliomas, which further enrich our understanding to glioma.

## Conclusions

This study firstly compared the protein expression of SOX2 in primary and its paired recurrent HGG. The expression of SOX2 decreased in recurrent gliomas compared with the primary gliomas. Lower SOX2 expression was seen in those patients who received adjuvant therapy. Low expression of SOX2 in primary HGG predicts poor prognosis. Those patients with SOX2 expression decreased in recurrent glioma often had worse prognosis.

## Supplementary information

**Additional file 1: Table S1.** SOX2 mRNA expression in 153 glioma cases in the TCGA database.

## Data Availability

The datasets used and analysed during the current study are available from the corresponding author on reasonable request.

## Abbreviations

DAB: Diaminobenzidine
FFPE: Formalin-Fixed and Parrffin-Embedded
GBM: Glioblastoma multiforme
HGG: High grade glioma
LGG: Low grade glioma
OS: Overall survival
RT: Radiotherapy
SAHZU: Zhejiang University School of Medicine
SOX2: SRY-related HMG-box 2
TCGA: The Cancer Genome Atlas
TMZ: Temozolomide
